# Co-expression of BirA with biotin bait achieves *in vivo* biotinylation of overexpressed stable *N*-glycosylated sRAGE in transgenic silkworms

**DOI:** 10.1038/s41598-017-00420-4

**Published:** 2017-03-23

**Authors:** Miyuki Kumano-Kuramochi, Ken-ichiro Tatematsu, Mayumi Ohnishi-Kameyama, Mari Maeda-Yamamoto, Toshiro Kobori, Hideki Sezutsu, Sachiko Machida

**Affiliations:** 1 Food Research Institute, NARO, 2-1-12 Kannondai, Tsukuba, Ibaraki 305-8642 Japan; 2 Institute of Agrobiological Sciences, NARO, 1-2 Owashi, Tsukuba, Ibaraki 305-8643 Japan

## Abstract

Here, we demonstrated the expression of the *N*-glycosylated extracellular ligand binding domain of receptor for advanced glycation end products (sRAGE) in middle silk glands (MSGs) of transgenic silkworms using the GAL4/UAS system. Over 1 mg of sRAGE was obtained from one transgenic silkworm. sRAGE purified from the silkworm exhibited good stability and maintained specific ligand-binding ability. In addition, *N*-glycan analysis of sRAGE revealed that *N*-glucan completely lacked potentially allergenic fucose. Moreover, co-expression of biotin ligase (BirA) with C-terminal BioEase-tagged sRAGE in MSGs resulted in efficient biotinylation of sRAGE after addition of biotin bait. C-terminal biotinylated sRAGE could be immobilized onto a solid surface in one direction through binding to streptavidin without any loss of ability. The dissociation constant of sRAGE with fructose-BSA, a typical RAGE ligand, was 7.25 × 10^−7^ M, consistent with that on the mammalian cell surface. Thus, we developed a novel, innovative silkworm expression system for efficient expression of recombinant sRAGE, which could serve as a basis for the elucidation of RAGE-ligand interactions and facilitate the search for new ligands and inhibitors.

## Introduction

The receptor for advanced glycation end products (RAGE) was originally identified as a receptor for advanced glycation end products (AGEs) and has been shown to be involved in the pathogenesis of diabetic complications. RAGE belongs to the immunoglobulin superfamily and is composed of three immunoglobulin-like domains (one V-type domain and two C-type domains), a short transmembrane region, and a signal transducing cytoplasmic domain^1, 2^. Recent studies have shown that RAGE is expressed in multiple cell types and functions as a pattern recognition receptor, binding structurally diverse ligands, such as amyloid-β, members of the S100 protein superfamily, and high-mobility group box-1 protein (HMGB-1)^3–5^. Ligand binding to RAGE can activate multiple signaling cascades^6, 7^, including those linked to Alzheimer’s disease, chronic inflammation, and cancer^8–10^. RAGE is expressed at low concentrations under normal physiological conditions in adults but is upregulated in the presence of its ligand and overexpressed in most affected tissues in RAGE-related pathogenic conditions^6, 10, 11^. Thus, RAGE has attracted much attention as a potential therapeutic target^12, 13^.

In addition to the soluble form of RAGE, an extracellular ligand-binding domain of RAGE (sRAGE)^14^ and a secreted splice variant (endogenous secretory RAGE [esRAGE]) have also been reported^15^. esRAGE is thought to function as a decoy receptor and bind AGEs without activating cellular signaling, whereas sRAGE blocks receptor activation in mouse models of various diseases^16, 17^. Currently, sRAGE has been expressed and purified from HEK293, *Escherichia coli*, Baculovirus, and yeast *Pichia pastoris* expression systems^2, 18, 19^. However, these expression systems do not yield sufficient amounts of sRAGE, mostly due to folding problems, protein instability, and high cost.

Transgenic silkworm technology has recently been investigated as a potential new protein expression system^20, 21^. The middle silk glands (MSGs) of silkworms (*Bombyx mori*) can adapt to produce robust amounts of sericin, demonstrating the capacity of this organ for expression of large amounts of recombinant protein in the soluble form using the GAL4/UAS system^21, 22^. Furthermore, protein expressed in MSGs may be glycosylated, which is expected to enhance the stability of the target protein. Additionally, *N*-glycans generated in MSGs include only oligo mannose and β-*N*-acetylglucosamine (GlcNAc), without the potentially allergenic core α 1–3 fucose^20, 22, 23^.

Therefore, in the present study, we examined the potential application of transgenic silkworms as an expression system for sRAGE. Our results demonstrated the production of *N*-glycosylated, biotinylation domain-tagged sRAGE in MSGs of transgenic silkworms, facilitating biotinylation after co-expression of biotin ligase (BirA) and addition of biotin bait.

## Results

### Expression of human sRAGE in the MSGs of transgenic silkworms

**Figure 1:**
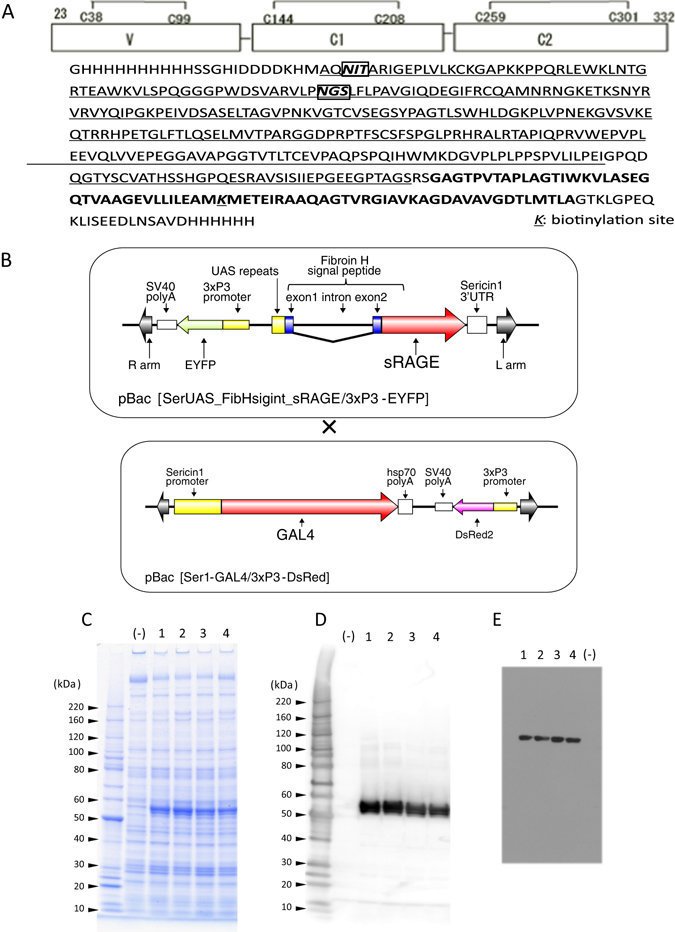
Expression of sRAGE in MSGs of transgenic silkworms. (**A**) Upper panel: Schematic diagrams of the domain structure of the extracellular domain of human RAGE. Numbers correspond to the amino acid (aa) sequence of full-length hRAGE, a 404-amino acid protein composed of an extracellular domain (aa 23–332). C represents cysteine residues involved in S-S linkages. Lower panel: Amino acid sequence of sRAGE expressed in this study. Underlined amino acids represent the RAGE region; bold indicates the BioEase tag; and boxes represent the potential *N*-glycosylation sites. (**B**) Generation of transgenic silkworms expressing the *sRAGE* gene. Adults transgenic silkworms harboring the *sRAGE* gene or *GAL4* gene were mated. The MSGs were isolated from F1 larvae, and total proteins were extracted. (**C**) CBB staining of SDS-PAGE. The protein lysates extracted from MSGs were separated by SDS-PAGE followed by staining with CBB. (**D**) Detection with anti-His-tag antibodies. Lysates were separated with SDS-PAGE, followed by western blotting with anti-His-tag antibodies. The protein lysates extracted from MSGs of transgenic silkworms harboring only the Ser1-GAL4 construct were used as negative controls (−). (**E**) Detection with anti-RAGE antibodies. The numbers above the gel or blots indicate the line number of the transgenic strain. Numbers on the left indicate the molecular mass (kDa).

The His-tagged extracellular domain of human RAGE composed of V, C1, and C2 domains (Fig. 1A), which represents the domain responsible for ligand recognition of RAGE, was expressed in MSGs using the GAL4/UAS system. To establish transgenic silkworm strains expressing sRAGE, we constructed a *piggyBac* vector harboring the *sRAGE* gene under control of UAS repeats. This vector was injected into silkworm eggs, and G1 embryos were screened for the expression of the *EYFP* gene in the eyes. Five broods that contained positive eggs were obtained, and four transgenic silkworm lines were established. To express the *sRAGE* gene in MSGs of transgenic silkworms, these transgenic silkworm lines were mated with the Ser1-GAL4 strain^22^, which expressed the *GAL4* gene in MSGs. In the next generation, the transgenic silkworms that expressed both EYFP and DsRed2 in embryonic eyes were selected and reared (Fig. 1B). MSGs were isolated from the fifth instar larvae and solubilized with phosphate-buffered saline (PBS) (−) containing 1% Triton X-100. The clear expression of a protein having the appropriate molecular weight of sRAGE was observed by sodium dodecyl sulfate polyacrylamide gel electrophoresis (SDS-PAGE) followed by Coomassie staining (Fig. 1C). The overexpressed proteins were analyzed by western blotting using both anti-His-tag antibodies (Fig. 1D) and anti-RAGE antibodies (Fig. 1E). No signal was detected in the negative control, whereas strong signals were detected in all lines in which the expression of sRAGE in MSGs was under the control of the GAL4/UAS system. These data suggested that the GAL4/UAS system could be used for expression of human sRAGE in MSGs of transgenic silkworms. Solubilized protein was purified by TALON metal affinity resin, as described in Materials and Methods, and over 1 mg of sRAGE was purified from only one silkworm. Furthermore, overexpression of sRAGE does not affect larvae development and the immune system because of remarkable location-specific expression^22^.

**Figure 2:**
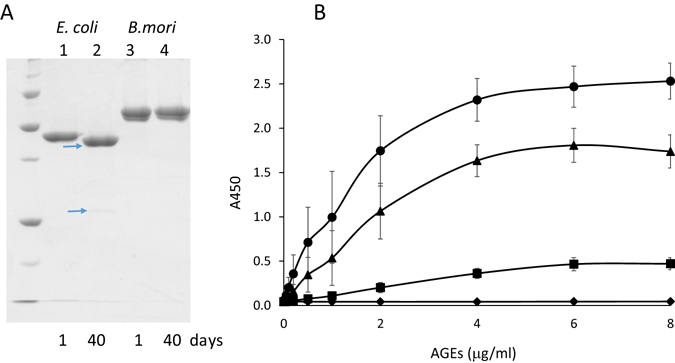
Characterization of sRAGE from transgenic silkworms. (**A**) Stability of sRAGE during storage at 4 °C. Forty days after purification, 10 μg of protein was applied to SDS-PAGE followed by CBB staining. Lanes 1 and 2: sRAGE expressed in *E. coli*; lanes 3 and 4: sRAGE expressed in MSGs of transgenic silkworms. Lanes 1 and 3: 1 day storage; lanes 2 and 4: 40 days of storage. Arrows indicate the degraded sRAGE. (**B**) Binding ability of sRAGE from silkworms to different AGEs after 1 year of storage. ⦁: fructose-AGEs; ▴: ribose-AGEs, ◼: glucose-AGEs, ⬧: control BSA (without glycation). Values are means and SEs of three wells from three independent experiments.

We next evaluated the stability of sRAGE expressed in silkworms. sRAGE produced using an *E. coli* system resulted in degradation within 40 days^24^; in contrast, sRAGE produced in silkworms was stable (Fig. 2A). To further explore the ligand recognition properties of sRAGE, we investigated whether sRAGE from silkworms could recognize model ligands. Importantly, sRAGE could recognize representative AGEs and did not react with nonglycosylated bovine serum albumin (BSA; Fig. 2B). In addition, sRAGE produced in transgenic silkworms could recognize representative AGEs even after 1 year. Furthermore, the binding affinity of sRAGE to each sugar-derived AGEs was different, with relative binding affinities in the order of fructose > ribose > glucose, consistent with that expressed in *E. coli* (Fig. S1). These results suggested that sRAGE from transgenic silkworms maintained its specificities for various sugar-derived AGEs. Additionally, sRAGE could recognize HMGB-1, another type of ligand, as well as sugar-derived AGEs (Fig. S2).

### Identification of glycosylation sites in sRAGE expressed in the MSGs of transgenic silkworms

**Figure 3:**
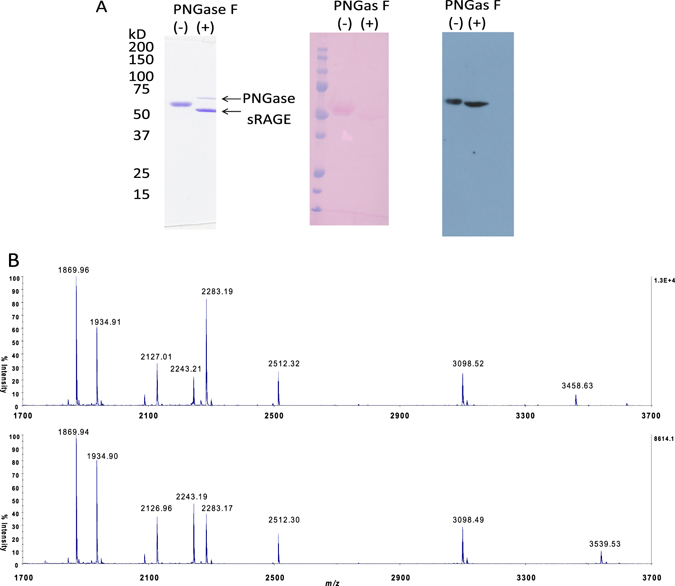
Analysis of glycosylation. (**A**) Left panel: CBB staining of 12% gels of sRAGE with (+) or without (−) PNGase treatment. Central panel: glycoprotein staining. sRAGE proteins with or without PNGase treatment were separated with SDS-PAGE, followed by blotting to nitrocellulose membranes and glycoprotein staining. Right panel: detection with anti-RAGE antibodies. (**B**) MALDI-TOF mass spectra of trypsin-digested sRAGEs without (upper panel) and with PNGase F treatment (lower panel). The observed peaks were as follows: *m/z* 1870.0 for AVSISIIEPGEEGPTAGSR (315–333), *m/z* 2127.0 for LISEEDLNSAVDHHHHHH (416–433) and HPETGLFTLQSELMVTPAR (180–198), *m/z* 2243.2 for VLP**N**GSLFLPAVGIQDEGIFR (78–98 deamidation) and VLASEGQTVAAGEVLLILEAMK (351–372 deamidation), *m/z* 2283.2 for RHPETGLFTLQSELMVTPAR (179–198, deamidation), *m/z* 2512.3 for VYQIPGKPEIVDSASELTAGVPNK (117–140), and *m/z* 3539.5 for GHHHHHHHHHHSSGHIDDDDKHMAQ**N**ITAR (0–29, deamidation).

Glycosylation contributes to the proper folding and stability of proteins^25, 26^. Therefore, we next investigated whether sRAGE expressed in silkworms was glycosylated. A shift in the molecular mass of sRAGE was observed after PNGase F treatment. The signal resulting from glycoprotein staining was absent after PNGase F treatment. On the other hand, the signal detected by anti-sRAGE antibodies was observed both before and after PNGase F treatment (Fig. 3A). Additionally, the molecular mass shift was confirmed by analysis of the mass spectra (Fig. S3). These results clearly showed that sRAGE expressed in transgenic silkworms was glycosylated.

Next, we sought to identify the glycosylation site in sRAGE. There are two putative glycosylation sites with sequences of N-X-S/T, N^25^IT, and N^81^GS in the V domain of sRAGE (Fig. 1A). We focused on the Asn-containing peptides generated by removal of *N*-linked glycans. Tryptic digestion of gel-purified sRAGE was analyzed by both matrix-assisted laser desorption/ionization (MALDI)-time-of-flight (TOF) and LC-electrospray ionization (ESI) mass spectrometry.

As shown in Fig. 3B, the ion peak at *m*/*z* 3539.5 was observed only in the MALDI-TOF mass spectrum for the *N*-glycan-removed peptides with the sequence of GHHHHHHHHHHSSGHIDDDDKHMAQNITAR (G^0^-R^29^) containing N^25^IT as the deamidated form at *m*/*z* 3539.5 (lower panel) but not in that for the peptide G^0^-R^29^ without PNGase F treatment (upper panel). Additionally, in LC-ESI MS/MS, the peptide HMAQNITAR (H^21^-R^29^) containing N^25^IT was observed as HMAQDITAR only in the PNGase F-treated sample (Fig. S3A,B). The PNGase F cleavage breaks the bond between GlcNAc and the glycosylated Asn residue, resulting in the conversion of Asn to Asp leading to a 1-Da mass increase. This result suggested that the peptide H^21^-R^29^ had an *N*-glycan at N^25^.

The nonglycosylated and the *N*-glycan-removed peptides with the sequence of VLPNGSLFLPAVGIQDEGIFR (V^78^-K^98^) containing N^81^GS were expected to give [M + H]^+^ at *m/z* 2242.22 (for the deamidated form at *m/z* 2243.21) and *m/z* 2243.21, respectively. Here, the peptide V^351^LASEGQTVAAGEVLLILEAMK^372^ (V^351^-K^372^) having a molecular mass similar to that of V^78^-K^98^ was expected to be observed at *m/z* 2242.24 in the samples with and without PNGase F treatment, because it did not have *N*-glycans. Indeed, ion peaks at *m*/*z* 2243.2 were observed in the mass spectra of both samples for the deamidated V^351^-K^372^. The relative intensity of the PNGase F-treated sample was higher (Fig. 3B, lower panel) than that of non-PNGase F-treated sample (Fig. 3B, upper panel) compared with other peptide peaks at *m*/*z* 2127.0, *m*/*z* 2512.3, and *m*/*z* 3098.5. These results were confirmed by LC-ESI MS (Fig. S4). Hence, we concluded that sRAGE was glycosylated at both Asp25 and Asp81.

### Structure determination of N-glycans

To gain insights into the *N*-glycosylation properties of sRAGE expressed in MSGs of transgenic silkworms, we determined the structures of *N*-glycans using a two-/three-dimensional (2D/3D) high-performance liquid chromatography (HPLC) mapping technique with “glycoanalysis by the three axes of MS and chromatography (GALAXY)”. The reducing ends of the released *N*-glycans from sRAGE were fluorescently labeled with 2-aminopyridine (2-PA), and the PA-glycan mixture was separated on a DEAE column and further purified using an ODS column, resulting in four peaks (N1, N2, N3, and N4; Fig. S5). The elution time on the ODS column of each glycan was expressed as glucose units (GU) to be plotted on the X-axis of the 2D map (Fig. S7). Next, each PA-glycan separated on the ODS column was applied onto the amide column. ODS peaks N1, N2, and N3 were combined into a single peak on the amide column, and ODS peak N4 was separated into three peaks (N4-1, N4-2, and N4-3; Fig. S6). The elution time was expressed as GU to be plotted on the Y-axis of the 2D map (Fig. S7). The structure of each *N*-glycan was estimated using GALAXY, and candidates were chosen (Fig. S7). Finally, the sample PA-glycan and the candidate reference of PA-glycan were co-injected into the ODS column to confirm the presence of a single peak (Fig. S8). The structures of the *N*-glycans are summarized in Supporting Table S1. The major glycans were identified as small mannose glycans (N1, N2, and N3), and these glycans represent nearly 90% of the total glycans (Table 1). A small portion of glycan contained GlcNAc on the Manα1-3 branch. There were no fucose, galactose, or sialic acid residues.

**Table 1:** Compositions and quantitative values of the identified *N*-glycans.

ODS peak No.	*N*-glycan	Composition ratio (%)	Quantitative value (pmol/mg)	Sugar composition
N1 + N1′	N1	27.5	3145	(Man)7(GlcNAc)2(PA)1
N2	N2	10.8	12131	(Man)6(GlcNAc)2(PA)1
N3 + N3′	N3	51.4	5872	(Man)5(GlcNAc)2(PA)1
N4	N4-1	2.8	315	(Man)3(GlcNAc)3(PA)1
	N4-2	4.6	525	(Man)3(GlcNAc)3(PA)1
	N4-3	2.5	291	(Man)4(GlcNAc)3(PA)1

### Co-expression of BirA in MSGs and addition of biotin bait

**Figure 4:**
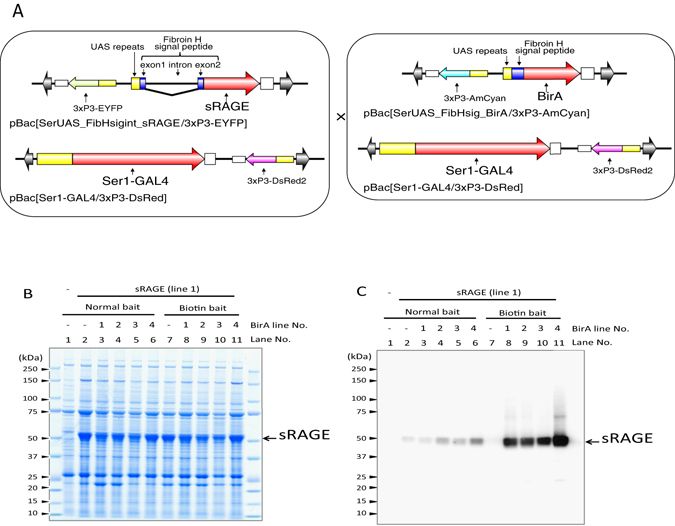
Effects of co-expression of the *BirA* gene and biotin bait on sRAGE biotinylation. (**A**) Generation of transgenic silkworms expressing both the *sRAGE* and *BirA* genes. Adult transgenic silkworms expressing the *sRAGE* gene or *BirA* gene were mated. During the fifth instar, approximately 20 μg/g biotin bait was fed to the larvae. The MSGs were isolated from the F1 larvae, and total proteins were extracted. (**B**) CBB staining of SDS-PAGE gels. Proteins extracted from MSGs were separated by SDS-PAGE followed by staining with CBB. (**C**) Detection with streptavidin-HRP. Lysates were separated by SDS-PAGE, followed by western blotting with streptavidin-HRP. Protein extracted from MSGs of transgenic silkworms harboring only the Ser1-GAL4 construct was used as a negative control (lane 1). Numbers at the left indicate molecular masses (kDa).

The sRAGE sequence was designed to add a BioEase tag (Fig. 1A), which was expected to be expressed in the biotinylated form in MSGs of transgenic silkworms. The BioEase tag is a 72-amino acid fragment derived from the oxaloacetate decarboxylase α subunit of *Klebsiella pneumoniae* and carries a single biotin-binding site^27^. The BioEase tag sequence is thought to be sufficient to facilitate *in vivo* biotinylation of the recombinant protein in mammalian and insect cells^28, 29^; however, the biotinylation efficiency of sRAGE produced in MSGs was below the detect limit by western blotting followed by incubation with horseradish peroxidase (HRP)-streptavidin. Therefore, we established a transgenic silkworm line co-expressing the biotin ligase BirA^30^ and sRAGE in MSGs (Fig. 4A). To generate transgenic silkworm strains expressing BirA, we constructed a *piggyBac* vector that harbored the *BirA* gene under the control of UAS repeats. The established transgenic silkworm lines were mated with the Ser1-GAL4 strain. To express both the sRAGE and the BirA in one larvae, the sRAGE-expressing line (line 1 in Fig. 1C) and BirA-expressing lines were mated, and transgenic silkworms that expressed EYFP, AmCyan, and DsRed2 in the eyes were selected and reared. When MSGs were isolated and solubilized with solubilization buffer (PBS (−) containing 1% Triton X-100), clear expression of sRAGE was observed by SDS-PAGE followed by CBB staining (Fig. 4B). However, the effects of co-expression of BirA were limited (Fig. 4C, lanes 3–6). Next, we added bait containing high levels of biotin (approximately 20 μg/g bait) to fifth stage instar larvae. The addition of this biotin bait resulted in efficient biotinylation of overexpressed sRAGE (Fig. 4C, lanes 8–11). Thus, co-expression of BirA and sRAGE plus addition of biotin bait was an effective method for production of biotinylated protein using transgenic silkworm technology.

### Kinetic analysis of the binding between sRAGE and AGEs

Biotinylated sRAGE was purified by TALON metal affinity resin followed by a streptavidin mutein matrix. Nearly 60% of sRAGE was biotinylated, and over 0.3 mg of biotinylated sRAGE was purified from one transgenic silkworm (Fig. S9). To further explore the advantage of biotinylation, we investigated whether the biotinylated sRAGE could function on a solid surface after immobilization through streptavidin using surface plasmon resonance (SPR). Immobilization of the target protein was performed by amine coupling and analyzed with a CM5 sensor chip^31^. Although no special peptide sequence is required with CM5, it is impossible to regulate the direction of immobilized protein, and in some cases, amine coupling causes loss of protein function.

**Figure 5:**
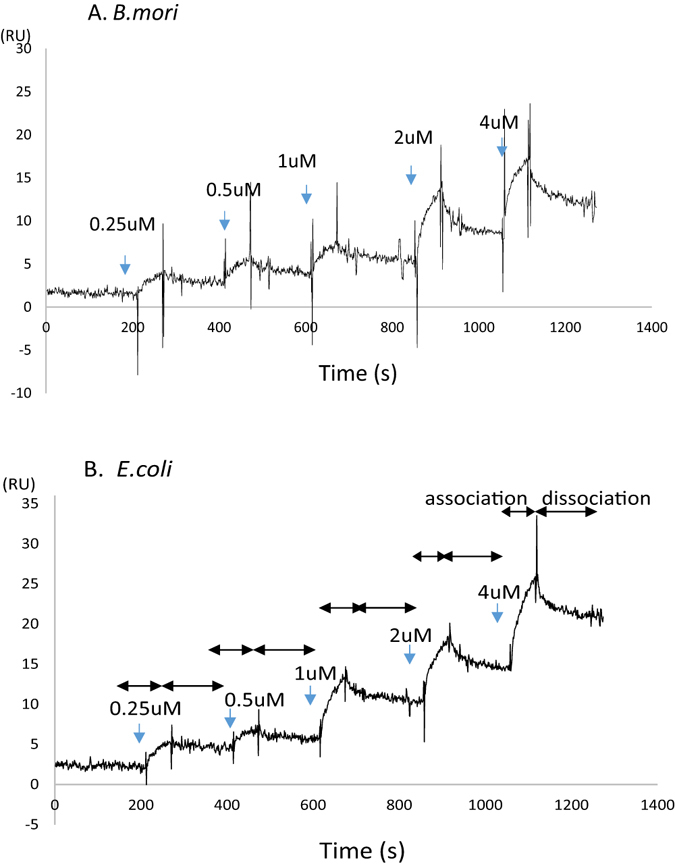
Sensorgrams of specific binding of serially diluted ligands to immobilized sRAGE on an SA sensor chip. Sequentially diluted ligands (0.25, 0.5, 1, 2, and 4 μM) were injected at the points indicated by arrows. (**A**) sRAGE expressed in *B. mori*. (**B**) sRAGE expressed in *E. coli*. Ligand (F-AGEs) diluted with running buffer (PBS) was injected over flow cells at a flow rate of 30 μL/min to allow 1 min association, and the flow cell was allowed 2 min for dissociation using a single-cycle kinetics program. Two reference curves (one curve derived from the injection of buffer only, and one curve derived from nonimmobilized sRAGE) were subtracted from the binding curve to exclude the effects of nonspecific binding. The dissociation constant of ligands to sRAGE was calculated based on the 1:1 (Langmuir) binding model.

When sRAGE was immobilized to CM5 using amine coupling, sensorgrams showed little response, even after injection of high concentrations of F-AGE (Fig. S10). Next, we examined the interactions of AGEs and sRAGE using an SA chip, which immobilized biotinylated sRAGE through streptavidin without any chemical treatment. In contrast to the experiments using CM5, sensorgrams (Fig. 5A) showed an increase in the RU reflective of AGE binding (association) and a slow decrease in response consistent with loss of mass from washout (dissociation) after each injection. We analyzed the sensorgrams by fitting them with a simple 1:1 Langmuir model. The K_D_ value was calculated to be 7.25 × 10^−7^ M (the association rate constant, k_a_, was 7.25 × 10^3^ M^−1^s^−1^ [SE (k_a_): 321], and the dissociation rate constant, k_d_, was 1.65 × 10^−3^ s^−1^ [SE (k_d_):1.5 × 10^−4^]), which was similar to that on the cell surface^1, 2, 32^, and a slow dissociation was also observed. The χ^2^ value of 1.51 indicated an adequate fit of the model to the data (<10: good fit; <2: ideal fit). Furthermore, the SE was less than 10%, and the data fitting results were sufficient. These parameters (χ^2^ and SE) indicated reliable data. We analyzed the interaction between F-AGEs and biotinylated sRAGE produced from *E. coli* and transgenic silkworms (Fig. 5B). The K_D_ value was 2.8 × 10^−7^ M, the k_a_ was 8.61 × 10^3^ M^−1^s^−1^ (SE: 153), and the k_d_ was 2.41 × 10^−3^ s^−1^ (SE: 8.08 × 10^−5^). In this case, the χ^2^ value, which reflects the closeness in the least-squares fitting, was 0.269. No significant differences in the association and disassociation rates were observed between *E. coli* sRAGE (without *N*-glycan) and transgenic silkworm sRAGE (*N*-glycosylated).

## Discussion

In this study, we aimed to develop a protein expression system to produce large amounts of sRAGE exhibiting high stability and activity. Our results showed that transgenic silkworms could be used as a model system to express biotinylated sRAGE, which could then be used in various applications.

RAGE interacts with many functionally and structurally different ligands and activates a variety of signal transduction cascades, supporting its potential applications as a therapeutic target^11, 12^. In order to elucidate the interactions between RAGE and its ligands, the recombinant ligand recognition domain of RAGE has been produced^17–19^. Of the three domains of RAGE^1, 2^, the V and C1 domain represent an integrated structural unit, whereas the C2 domain is independent^14^. A recent study indicated that most ligands, such as AGEs, HMGB-1, and aβ peptides bind to the V domain^33, 34^; some ligands, such as S100A12, bind to the V-C1 domain^35^, whereas S100A6 interacts with the V-C2 domain^36^. It is difficult to prepare recombinant sRAGE (V-C1-C2) as a stable form^24^. Therefore, many studies have focused on the interaction between the ligand and the recombinant V domain or V-C1 domain. Additionally, esRAGE and sRAGE have been reported to function as decoy receptors, binding to AGEs without activating cellular signaling^14–17^. Thus, in this study, we developed a method for expressing sRAGE (full-length extracellular domain composed of V, C1, and C2 domain) for use in various applications.

Although sufficient amounts of active sRAGE have been produced using an *E. coli* system^19^, the sRAGE produced in *E. coli* is not stable and is degraded within 40 days. This lack of stability may be explained by the lack of *N*-glycosylation, a common post-translation modification that modulates both the physiological and biological properties of proteins^25, 26^. RAGE has two *N*-glycosylation sites on the V domain; both sites are occupied by complex and hybrid *N*-glycans^36^. Accordingly, in this study, we produced *N*-glycosylated sRAGE using transgenic silkworm technology. *N*-glycosylation significantly enhanced the stability of sRAGE, and this recombinant sRAGE maintained stability for over 1 year.

In the past decade, nonmammalian hosts have been used for the production of recombinant protein owing to the lower costs of protein expression and the ease of use of these hosts. *N*-glycosylated sRAGE production has been reported using HEK293 mammalian cells and the methylotrophic yeast *P. pastoris* ^18, 37^. The structures of *N*-glycans produced by *P. pastoris* are more similar to those of mammalian cells than the *N*-glycans produced by the budding yeast *Saccharomyces cerevisiae* ^38^. However, α1-2-linked galactose, which is generally absent in *N*-glycans expressed in mammalian hosts, may be abundant^39^. Moreover, in transgenic silkworms, the *N*-glycans often contain the potentially allergenic core α1-3-linked fucose (Fuc). However, researchers have overcome this problem by developing silkworms that produce recombinant protein in the MSG^22, 23^; the major glycans added onto glycoproteins produced in the MSGs are small mannosidic glycans and complex mannosidic glycans consisting GlcNAc and without α1-3 and α1-6 Fuc^20, 22, 23^. Thus, the sRAGE produced in MSGs in this study contained small mannose glycans as the primary glycan, with a small portion containing GlcNAc on the Manα1-3 branch; no Fuc was found. This highlights the advantages of our novel protein expression system for the humanization of *N*-glycans, resulting in therapeutically useful proteins for future applications.

Previous studies have indicated that the *N*-glycans on RAGE influence the binding of RAGE with several ligands, including HMGB-1 and S100A12^36, 40, 41^. However, the effects of *N*-glycans on AGEs had not previously been clarified. In this study, we found that sRAGE expressed in MSGs exhibited affinities similar to those of proteins expressed on the mammalian cell surface. Further studies are necessary to elucidate the effects of *N*-glycans on the interaction between RAGE and various ligands.

Recombinant proteins are usually expressed in heterogeneous hosts attached to the fusion tag, and biotin is one of the most useful tags for biochemical experiments because of its femtomolar dissociation constant with streptavidin^42^. Enzymatic attachment of biotin to proteins requires the interaction of a distinct domain (the biotinylation domain) in the acceptor protein with biotin ligase. Both biotin domains and biotin ligase are strongly conserved^42^. In this study, we attached a BioEase tag as an acceptor at the C-terminus of sRAGE; we expected that one biotin would be attached to the Lys residue in the BioEase tag, yielding biotinylated sRAGE^27^. However, the biotinylation efficiency of sRAGE expressed in MSGs was low. Biotinylation, catalyzed by biotin ligase, is expected to be the rate-determining step owing to the low expression of biotin ligase relative to the amount of overexpressed sRAGE. However, although co-expression of the biotin ligase BirA in MSGs increased the amount of biotinylated sRAGE, the biotinylation efficiency still was insufficient. Finally, we found that application of biotin bait to the fifth stage instar larvae resulted in dramatic improvement of biotinylation efficiency. Thus, our findings showed that co-expression of BirA in MSGs combined with application of biotin bait yielded a promising and efficient transgenic silkworm expression system. The biotinylated protein can be used in a variety of experiments, such as affinity selection, determination of protein interactions, and display of proteins on arrays.

## Materials and Methods

### Plasmid construction

To generate transgenic silkworms that express the *sRAGE* or *BirA* gene, *piggyBac* vectors harboring the *sRAGE* or *BirA* gene under the control of UAS repeats were constructed. As screening markers, the 3xP3-EYFP cassette and 3xP3-AmCyan cassette were used for *sRAGE* and *BirA*, respectively. The plasmid for generating transgenic silkworms expressing the *sRAGE* gene was prepared as follows. To remove the DNA fragment encoding the signal peptide of the *Sericin1* gene of pBac[SerUAS_Ser1intron_hr5/3xP3-EYFP_A3-Bla]^21^ and insert the silkworm kozak sequence into the plasmid^43^, the PCR fragment amplified from pBac[SerUAS-hr5/3xP3-EGFPinv] with primers SerTATA-U and BlnBsmSerK-L was digested with *SnaB*I and *BsmB*I and inserted into the *SnaB*I and *BsmB*I site of pBac[SerUAS_Ser1intron_hr5/3xP3-EYFP_A3-Bla]. The resulting plasmid was designated pBac[SerUAS_Ser1kozak_hr5/3xP3-EYFP_A3-Bla]. Table S2 summarizes the primers used in this study. To insert the signal peptide coding sequence of the fibroin H gene, the PCR fragment amplified from pHC-EGFP with primers FibHsig-U and FibHsig-L was digested with *BspH*I and *Bln*I, followed by insertion into the *BsmB*I and *Bln*I site of pBac[SerUAS_Ser1kozak_hr5/3xP3-EYFP_A3-Bla]. The resulting plasmid was named pBac[SerUAS_FibHsigint_hr5/3xP3-EYFP_A3-Bla]. The *sRAGE* gene was amplified from HisEK_hsRAGE1-BioEase-myc-His with primers BsmBI-bioRAGE-U and BsmBI-bioRAGE-L. The PCR fragment was digested with *BsmB*I and ligated into the *BsmB*I site of pBac[SerUAS_FibHsigint_hr5/3xP3-EYFP_A3-Bla] to generate pBac[SerUAS_FibHsigint_sRAGE/3xP3-EYFP].

The plasmid for generating transgenic silkworms expressing the *BirA* gene was constructed as described below. To change the fluorescent marker gene, the *AmCyan* gene was amplified from the pAmcyan1-N1 vector (TaKaRa) with the AmCyanKozak-U and AmCyan-L primers. The *Nco*I-*Not*I fragment of the PCR fragment was inserted into the *Nco*I-*Not*I site of pBac[SerUAS_Ser1kozak_hr5/3xP3-EYFP_A3-Bla] to generate pBac[SerUAS_Ser1kozak_hr5/3xP3-AmCyan_A3-Bla]. To insert the signal peptide sequence of the fibroin H gene, the FibHsigAd adapter (Table EV2) was inserted into the *BsmB*I-*Bln*I site of pBac[SerUAS_Ser1kozak_hr5/3xP3-AmCyan_A3-Bla]. The resulting plasmid was designated pBac[SerUAS_FibHsig_hr5/3xP3-AmCyan_A3-Bla]. The synthesized *BirA* gene (GenScript, Piscataway, NJ, USA) was inserted into the *BsmB*I-*Bln*I site of pBac[SerUAS_FibHsig_hr5/3xP3-AmCyan_A3-Bla] to generate pBac[SerUAS_FibHsig_BirA/3xP3-AmCyan].

### Generation of transgenic silkworms

Transgenic silkworms were generated as previously described^43, 44^ using the plasmids pBac[SerUAS_FibHsigint_sRAGE/3xP3-EYFP] and pBac[SerUAS_FibHsig_BirA/3xP3-AmCyan]. The silkworm strains were maintained at the Transgenic Silkworm Research Unit, National Institute of Agrobiological Sciences. Silkworm larvae were reared on an artificial diet (Nosan) at 25 °C.

### Expression of the *sRAGE* and/or *BirA* genes

Transgenic silkworm lines harboring the *sRAGE* or *BirA* gene under regulation of UAS repeats were mated with adults from the Ser1-GAL4 strain^23^, which carried the *GAL4* gene driven by the *Sericin1* promoter and a 3xP3-DsRed2 marker cassette. F1 embryos harboring both the GAL4 construct and the UAS construct were selected based on fluorescence of DsRed2 and EYFP or AmCyan. These F1 adults were mated with each other, and embryos that expressed DsRed2, EYFP, and AmCyan in their eyes were selected to obtain transgenic silkworms expressing both sRAGE and BirA.

### Oral administration of biotin bait to transgenic silkworms

Fifty milliliters of 0.2 mg/mL biotin (Wako) in 1 × PBS (−) was kneaded into 500 g of the artificial diet. Biotin containing an artificial diet was fed to fifth instar larvae of transgenic silkworms.

### Extraction of proteins from MSGs of transgenic silkworms

MSGs were isolated from larvae on the sixth day of the fifth instar and then immersed in 1 mL of 1 × PBS (−) containing 1% Triton X-100. Samples were shaken gently for 2 h at 4 °C. The resulting extract was frozen at −80 °C for 1 day and thawed at 4 °C; debris was removed from each extract by filtration.

### Purification of sRAGE from MSGs extracts

The crude extract was cleared by centrifugation, and *B. mori* sRAGEs were purified as follows. Cleared lysates were gently mixed with TALON (Clontech) resin slurry for 15 min at 4 °C. The resin was washed with PBS, and histidine-tagged proteins were eluted with PBS containing imidazole. For biotinylated sRAGE, samples were dialyzed against PBS and further purified through a streptavidin mutein matrix (Roche). A streptavidin mutein matrix slurry equilibrated with PBS was added to the dialyzed solution and gently mixed for 15 min at 4 °C, and biotinylated sRAGE was eluted with PBS containing d-biotin. Fractions containing protein at the expected molecular mass were collected, and their purity was analyzed by SDS-PAGE.

Biotinylated sRAGE in *E. coli* was purified by sequential chromatography steps using Ni-NTA resin and streptavidin mutein matrix, as described previously^19^ with modifications. Briefly, cleared lysate was mixed with Ni-NTA resin slurry for 15 min at 4 °C, and histidine-tagged proteins were eluted by FPLC with TBS containing 350 mM imidazole. Fractions containing protein were dialyzed against TBS and further purified through a streptavidin mutein matrix.

### SDS-PAGE and western blotting analyses

Protein lysates from MSGs were prepared and separated on 4–12% gradient gels (NuPAGE BisTris gel; Thermo Fisher Scientific) according to the manufacturer’s instructions or on 12% gels. For detection of the sRAGE protein, anti-His-tag antibodies (Bethyl Laboratories) or anti-RAGE antibodies (Santa Cruz Biotechnology) and HRP-conjugated anti-rabbit IgG antibodies (GE Healthcare) were used as the primary and secondary antibodies, respectively. A BenchMark protein ladder (Thermo Fisher Scientific) was used as the molecular marker. For detection of biotinylated sRAGE protein, a streptavidin-HRP conjugate (GE Healthcare) and Precision Plus Protein Kaleidoscope Standards (Bio-Rad) were used. Immunoreactive protein bands were detected with ECL prime (GE Healthcare) and an LAS-3000 image analyzer (Fuji Film).

### Preparation and detection of sugar-derived AGEs

Fifty microliters of sRAGE solution (5 μg/mL) was added to each microtiter well (Nunc) overnight at 4 °C. After the solution was discarded, the wells were washed three times with TBS containing 0.05% Tween 20 (TBS-T). The wells were then filled with 250 μL of nonprotein blocking buffer (Pierce) at room temperature for 2 h. After discarding the blocking solution, the wells were washed three times with TBS-T, 100 μL of AGE-BSA solution (0–8 μg/mL), prepared as previously described^19^, was applied to each well, and plates were incubated at room temperature for 2 h. The wells were then washed five times with TBS-T and incubated with 100 μL anti-BSA mouse monoclonal antibodies (Abcam; diluted 1:6000 with blocking buffer) for 1 h at room temperature. After washing the wells five times with TBS-T, the wells were incubated with 100 μL of anti-mouse IgG-HRP conjugate (Chemicon; diluted 1:5000 with blocking buffer) for 1 h at room temperature. After washing the wells five times with TBS-T, 50 μL of 3,3′,5,5′-tetramethylbenzidine (TMB) reactive substrate solution (Sigma) was added, and the plate was incubated for 20 min at room temperature. The enzyme reaction was stopped using 50 μL of 1 N HCl, and the absorbance at 450 nm was measured with a microplate reader (WALLAC ARVO SX; Perkin Elmer).

### PNGase treatment

Samples were incubated in G reaction buffer (New England Biolabs) with 2.5% Nonidet P-40 and 500 U PNGase F for 24 h at 37 °C. Glycoproteins were detected with a Glycoprotein Staining kit (cat. no. 24562; Thermo Fisher Scientific).

### MS analysis

Each sRAGE sample with or without PNGase F treatment was separated by SDS-PAGE. The bands on the gel containing sRAGE were cut out and applied to in-gel digestion with trypsin after reductive alkylation. Peptides produced by the tryptic digestion of samples were separated on an EASY Spray column (PepMapC18, 3 μm, 75 μm × 15 cm; Thermo Fisher Scientific) at 300 nL/min, with a gradient from 0% to 100% solvent B over 30 min. The solvents were as follows: A (0.1% formic acid in MilliQ water) and B (0.1% formic acid in acetonitrile). The eluate from the nanoLC apparatus (EASY nLC; Thermo Fischer Scientific) in line coupled to the nanospray source was analyzed on an Orbitrap Veros Pro (Thermo Fischer Scientific) mass spectrometer. For MALDI-TOF MS, the sample was spotted on a target plate with α-cyano-4-hydroxycinnamic acid (Bruker Daltonics) as the matrix. MALDI- TOF/TOF mass spectra were recorded on a 4800 plus MALDI TOF/TOF analyzer (AB SCIEX) in the positive-ion mode with an acceleration voltage of 20 kV. The mass spectrometers were tuned and calibrated using commercially available standard samples such as polytyrosine peptides (Thermo Fisher Scientific) and a peptide mixture kit (Peptide Calibration Standard II, Bruker Daltonics) prior to the measurements.

### 2D/3D HPLC mapping with GALAXY

2D/3D HPLC mapping with GALAXY was carried out with support from Medical and Biological Laboratories Co., Ltd. (Nagoya, Japan). sRAGE was denatured for 10 min at 100 °C, and the solvent was evaporated. The dried samples were digested with glycoamidase A and trypsin in 0.5 M citrate phosphate buffer (pH 4.0) overnight at 37 °C, followed by digestion with pronase in 1 M Tris-HCl buffer (pH 8.0) overnight at 37 °C. The carbohydrates and amino acids of the digestant were separated on a P-2 gel filtration column with MilliQ water. After confirmation of the presence of neutral sugars by orcinol-sulfuric acid methods, the carbohydrate fractions were collected and evaporated in vacuo. The samples were dissolved in coupling reagent (2-aminopyridine [2-PA] acetic acid solution) and fluorescently labeled with 2-PA for 60 min at 90 °C, followed by reduction with borane-dimethylamine complex. After drying, the PA-glycan mixture was separated on a Sephadex-G15 column (38 cm × 10 mm). PA-glycan fractions, sialylated sugars and neutral sugars were separated on a DEAE ion exchange column (7.5 mm × 7.5 cm) with a gradient from 0% to 100% solvent B over 28 min. The solvents were as follows: A, aqueous ammonia, pH 9.0; and B, 0.5 M ammonium acetate, pH 9.0. The neutral sugar fractions were collected and dried. The residue was redissolved in water and separated on an ODS column (6 mm × 15 cm) with a gradient from 0% to 100% solvent B over 60 min. The solvents were as follows: A, phosphate buffer, pH 3.8; and B, phosphate buffer containing 0.5% butanol, pH 3.8. The resulting fractions were analyzed by MALDI-TOF MS (matrix: 2.5% dihydroxybenzoic acid/50% acetonitrile) in order to confirm their identities as *N*-glycans. Next, each *N*-glycan was separated on an amide column (4.6 mm × 25 cm) with a gradient from 0% to 100% solvent B over 40 min. The solvents were as follows: A, 65% acetonitrile containing 2.9% triethylamine and 1.2% acetic acid; and B, 50% acetonitrile containing 4.1% triethylamine and 1.7% acetic acid. The structure of each *N*-glycan was estimated by both information in ODS and amide column chromatographies and mass analysis according to the GALAXY method. The sample PA-glycan and candidate reference N-glycan chosen by GALAXY were co-injected into the ODS column prepared under the condition mentioned above, and the profiles of the peak in the chromatogram were estimated.

### SPR assay

SPR assays were performed with a Biacore 2000 apparatus (GE Healthcare). Biotinylated sRAGE was immobilized on a CM5 sensor chip by amine-coupling according to the manufacturer or on an SA sensor chip (GE Healthcare) via the C-terminal biotin at a density of approximately 560 response units. The interaction between immobilized sRAGE and F-AGEs was examined at 25 °C with a flow rate of 30 μL/min using a single-cycle kinetics analysis program, and PBS (−) was used as running buffer. The response curves obtained from injecting buffer only and from the control flow cell (without immobilized sRAGE) were subtracted from the sRAGE-immobilized cell to correct for nonspecific binding. BIA evaluation software (version 4.1) was used to perform the analysis of the kinetics.

### Statistical analysis

For data quantification, three independent experiments were evaluated. Statistical analyses were performed with Microsoft Excel 2013.

## Electronic supplementary material

Supplementary Dataset 1
