# Supplementary material for: Co-expression of BirA with biotin bait achieves in vivo biotinylation of overexpressed stable N-glycosylated sRAGE in transgenic silkworms
Source: Sci Rep. 2017 Mar 23;7:356. doi: 10.1038/s41598-017-00420-4 (PMC5428419; doi:10.1038/s41598-017-00420-4)
Supplement: Supplementary file 1 — Supplementary Dataset 1 [file 41598_2017_420_MOESM1_ESM.doc]

**Supplementary Information**

**Co-expression of BirA with biotin bait achieves *in vivo* biotinylation of overexpressed stable *N*-glycosylated sRAGE in transgenic silkworms**

Miyuki Kumano-Kuramochi^1,✝^, Ken-ichiro Tatematsu^2,✝^, Mayumi Ohnishi-Kameyama^1^, Mari Maeda-Yamamoto^1^, Toshiro Kobori^1^, Hideki Sezutsu^2^ , Sachiko Machida^1,*^

^1^Food Research Institute, NARO, 2-1-12 Kannondai, Tsukuba, Ibaraki 305-8642, Japan

^2^The National Institute of Agrobiological Sciences, 1-2 Owashi, Tsukuba, Ibaraki, 305-8643, Japan

^✝^Both authors contributed equally to this work.

*Corresponding author

Authors’ emails

MKK: mkumano@affrc.go.jp

KT: K.Tatematsu@affrc.go.jp

MOK: kameyama@affrc.go.jp

MMY: marimy@affrc.go.jp

TK: tkobo@affrc.go.jp

TK: HS: hsezutsu@affrc.go.jp

SM: lili@affrc.go.jp

| Name | | Sequence |
| --- | --- | --- |
| primer | |  |
|  | SerTATA-U | 5'-GTACGTAAGCTTGATCAAACTTCGTTTTCG-3' |
|  | BlnBsmSerK-L | 5'-GCCTAGGGAGACGGCAGATCGTCTCCCATGTTGGCGGTCTTTGG-3' |
|  | FibHsig-U | 5'-GTCATGAGAGTCAAAACCTTTGTGATCTTG -3' |
|  | FibHsig-L | 5'-GCCTAGGGAGACGGCAGATCGTCTCCTGCATTTGTATAAGCGACA-3' |
|  | BsmBI-bioRAGE-U | 5'-GCGTCTCGTGCAGGCCATCATCATCATCATCATCATCATCATCACAGCAGCGGC-3' |
|  | BsmBI-bioRAGE-L | 5'-CCGTCTCGCTAGTTAATGATGATGATGATGATGGTCGACGGCGCT-3' |
|  | AmCyanKozak-U | 5'-CCATGGAAATCAAAATGGCCCTGTCCAACA-3' |
|  | AmCyan-L | 5'-GCGGCCGCTCAGAAGGGCACCACGGAGGTGAT-3' |
|  |  |  |
|  | FibHsigAd adapter | 5'-CATGAGAGTCAAAACCTTTGTGATCTTGTGCTGCGCTCTGCAGTATGTCGCTTATACAAATGCAGGAGACGATCTGCCGTCTCC-3' |
|  |  | 3'-TCTCAGTTTTGGAAACACTAGAACACGACGCGAGACGTCATACAGCGAATATGTTTACGTCCTCTGCTAGACGGCAGAGGGATC-5' |

Table S2. Primers used for plasmid construction


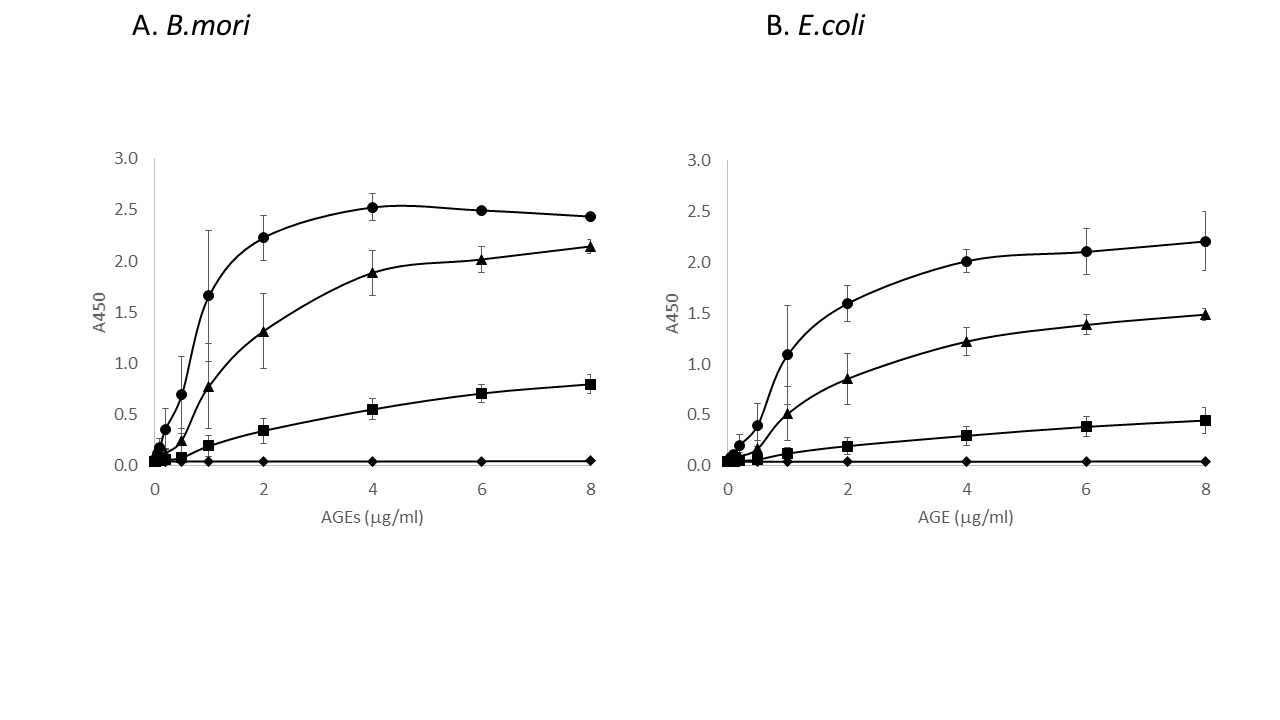


**Fig. S1** Binding ability of sRAGE from *B. mori* and *E. coli* to different AGEs just after purification. (A) Binding ability of sRAGE produced from *B. mori* after 1 day of storage. (B) Binding ability of sRAGE from *E. coli* after 1 day of storage. ●: fructose-AGEs, ▲: ribose-AGEs, ■: glucose-AGEs, ♦: control BSA (without glycation). Values are means of three wells from three independent experiments.

**Fig. S2** Binding ability of sRAGE from *B. mori* to HMGB-1. Fifty microliters of ligand solution was added to each microtiter well overnight at 4°C, and the wells were washed three times with TBS containing 0.05 % Tween 20 (TBS-T). Then wells were blocking with 250 μL of nonprotein blocking buffer at room temperature for 2 h. After discarding the blocking solution, the wells were washed three times with TBS-T, 100 μL of biotinylated sRAGE from *B.mori* (0.7 μg/mL) was applied to each well, and plates were incubated at room temperature for 1 h. The wells were then washed five times with TBS-T and incubated with 50 μL Streptavidin-HRP for 1 h at room temperature. After washing the wells five times with TBS-T, 50 μL of 3,3’,5,5’-tetramethylbenzidine (TMB) reactive substrate solution was added, and the plate was incubated for 20 min at room temperature. The enzyme reaction was stopped using 50 μL of 1N HCl, and the absorbance at 450 nm was measured with a microplate reader. Ligands, ●: fructose-AGEs, ■: HMGB-1. Values are means of three wells from two independent experiments.


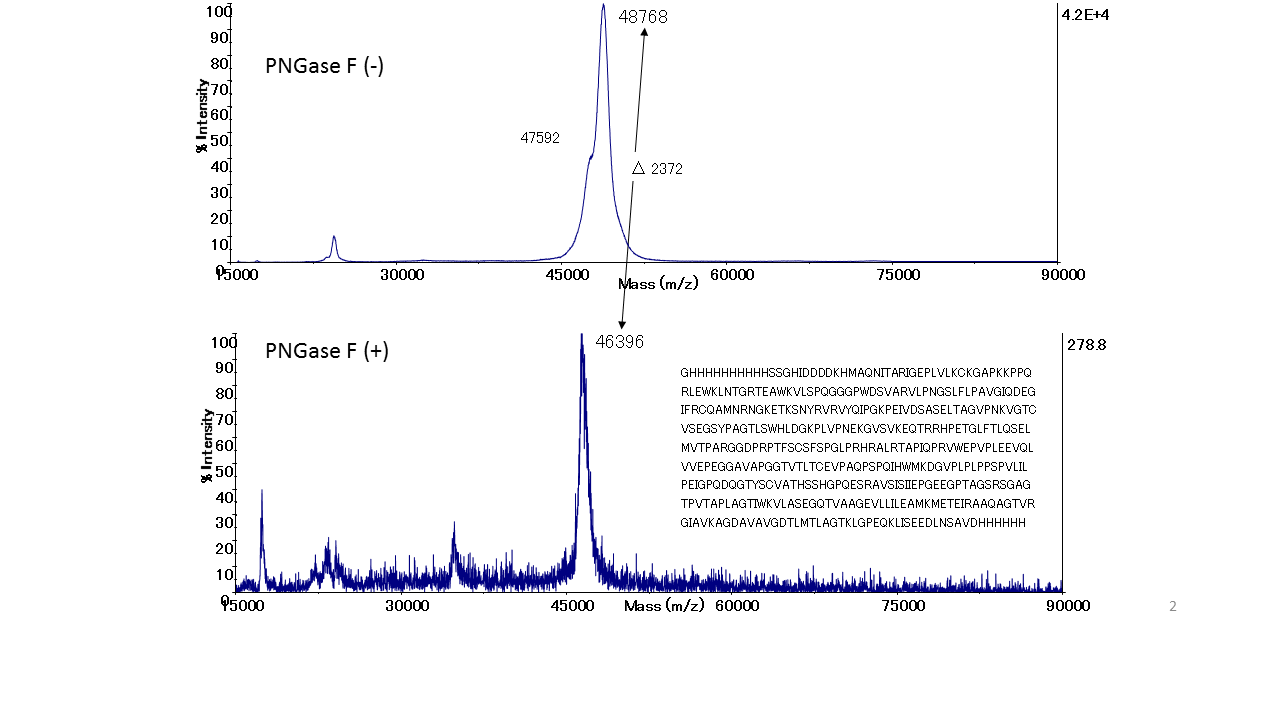


**Fig. S3** MALDI-TOF MS analysis of sRAGE with or without PNGase F treatment. Samples were spotted on MALDI target plates with α-cyano-4-hydroxycinnamic acid matrix and air dries. Spectra were acquired on a 4800 plus TOF/TOF Analyzer (AB SCIEX) using a nitrogen gas laser (337 nm). Upper panel: without PNGase F treatment, Lower panel: with PNGase F treatment.


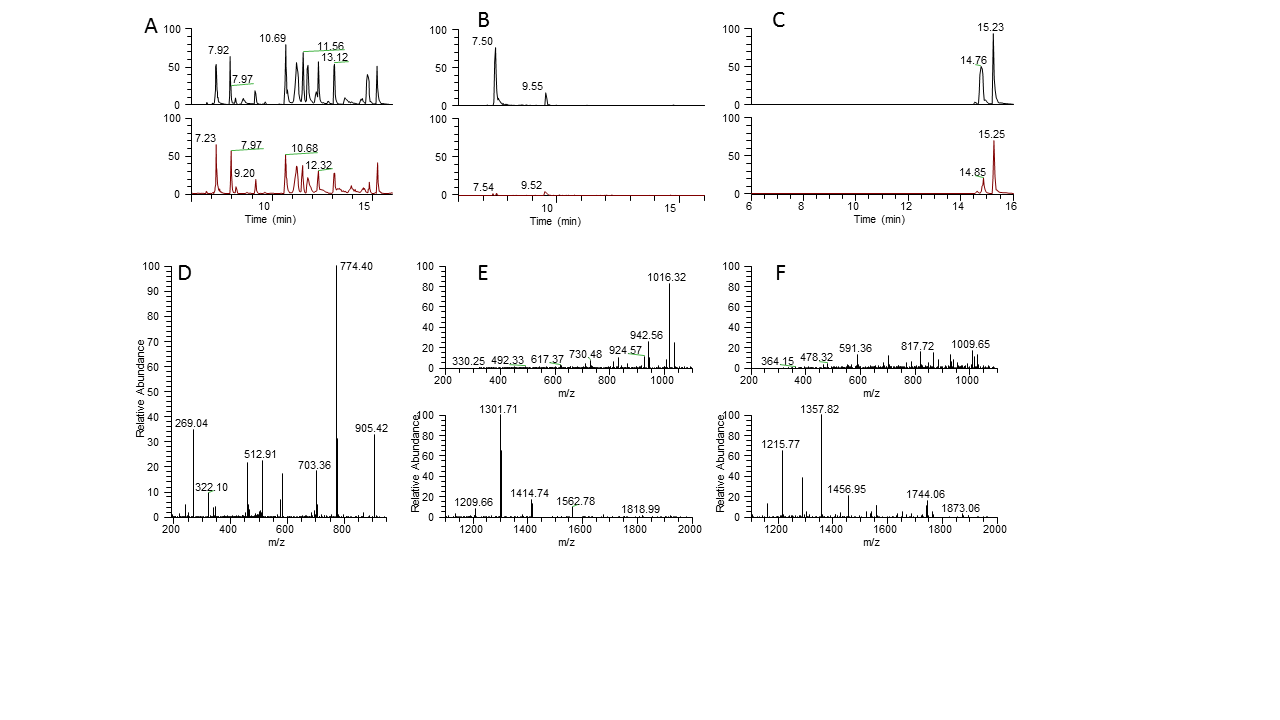


**Figure S4** LC- ESI-MS/MS analysis of sRAGE with or without PNGase F treatment. (A) The base peak chromatograms of sRAGE with (upper panel) and without PNGase F treatment (bellow panel) in the range of *m/z* 350-1500. (B) The extracted ion chromatograms of sRAGE with (above) and without PNGase F treatment (bellow) in the range of *m/z* 521.71-521.81 for HMAQ(N)ITAR (H^21^-R^29^) containing N^25^IT. (C) The extracted ion chromatograms of sRAGE with (above) and without PNGase F treatment (bellow) in the range of *m/z* 1122.06-1122.16 for VLP(N)GSLFLPAVGIQDEGIFR (V^78^-K^98^) containing N^81^GS. (D) Product ions from the peak at 7.5 min suggesting the amino sequence of HMAQ(N)ITAR. (E) Product ions from the peak at 14.8 min suggesting the amino sequence of VLP(N)GSLFLPAVGIQDEGIFR (V^78^-K^98^) containing N^81^GS. (F) Product ions from the peak at 15.2 min suggesting the amino sequence of V^351^-K^372^ having similar molecular mass to that of V^78^-K^98^. Here (N) means that the Asn residue is transformed to Asp after cleavage of the attached *N*-glycan.


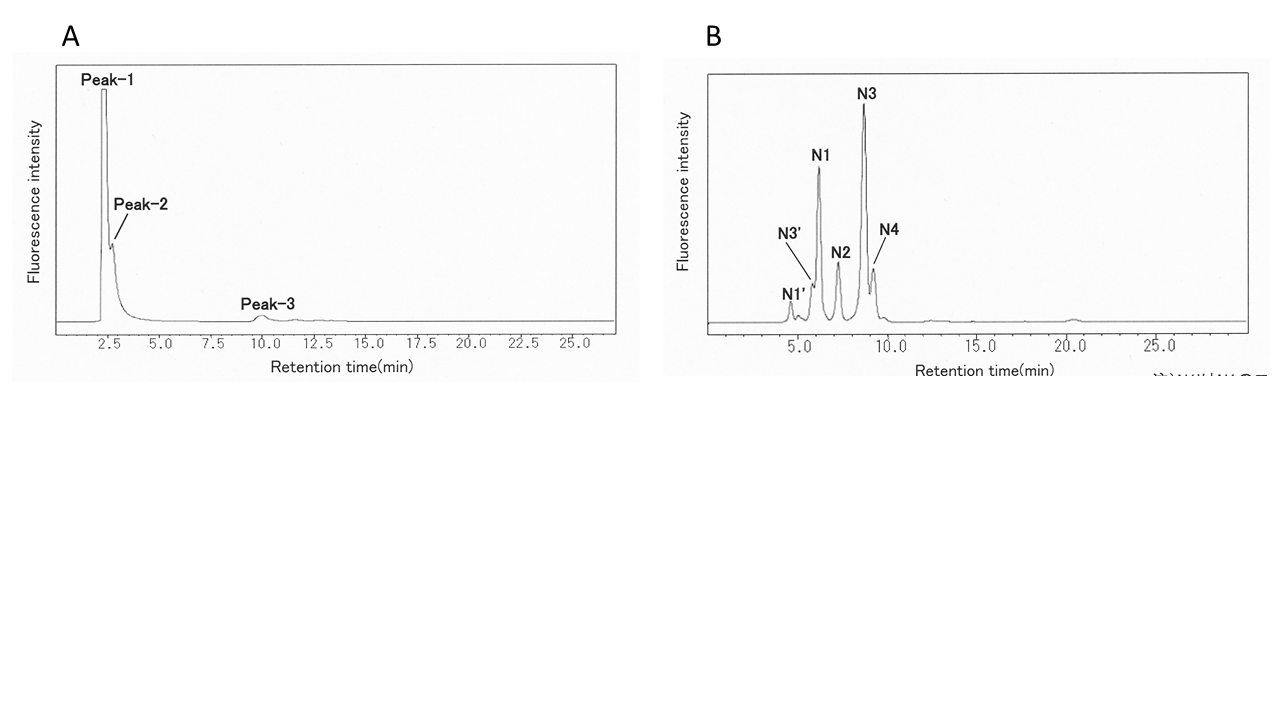


**Fig. S5** Separation of *N*-glycans from sRAGE expressed in MSGs of *B. mori.* *N*-glycans digested from sRAGE were fluorescently labeled with 2-aminopyridien (2-PA). (A) PA-glycans were separated on a DEAE column. Peak 1: neutral sugar fraction. Peaks 2 and 3 did not contain *N*-glycans. (B) DEAE peaks separated on an ODS column. DEAE peak 1 was separated into four peaks (N1+N1’, N2, N3+N3’, and N4). Each N1’ and N3’ peak was identical to the N1 and N3 peaks, respectively, because N1’ and N3’ were epimeru-sugar chains produced as byproducts during the 2-PA reaction.


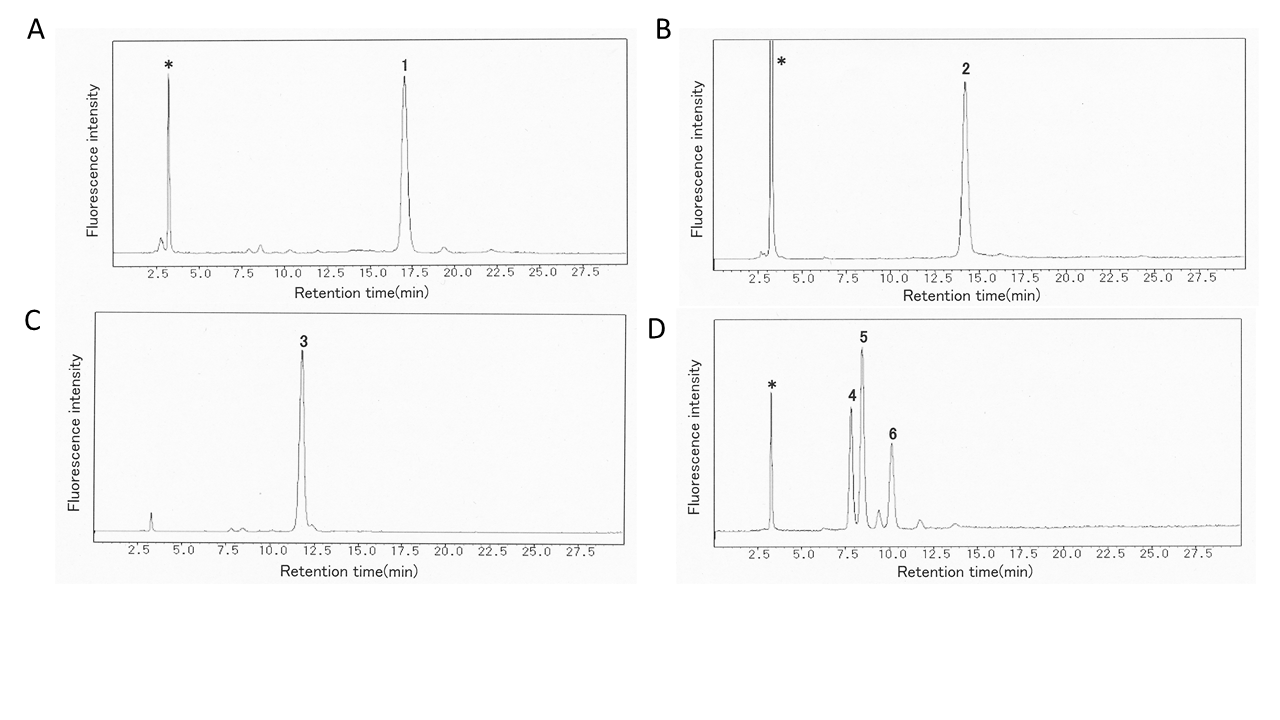


**Fig. S6** *N*-glycan separation using the Amide column. Each *N*-glycan peak was separated on the ODS column and further separated on the Amide column. (A) Peak N1 from the ODS column. (B) Peak N2 from the ODS column. (C) Peak N3 from the ODS column. (D) Peak N4 from the ODS column.


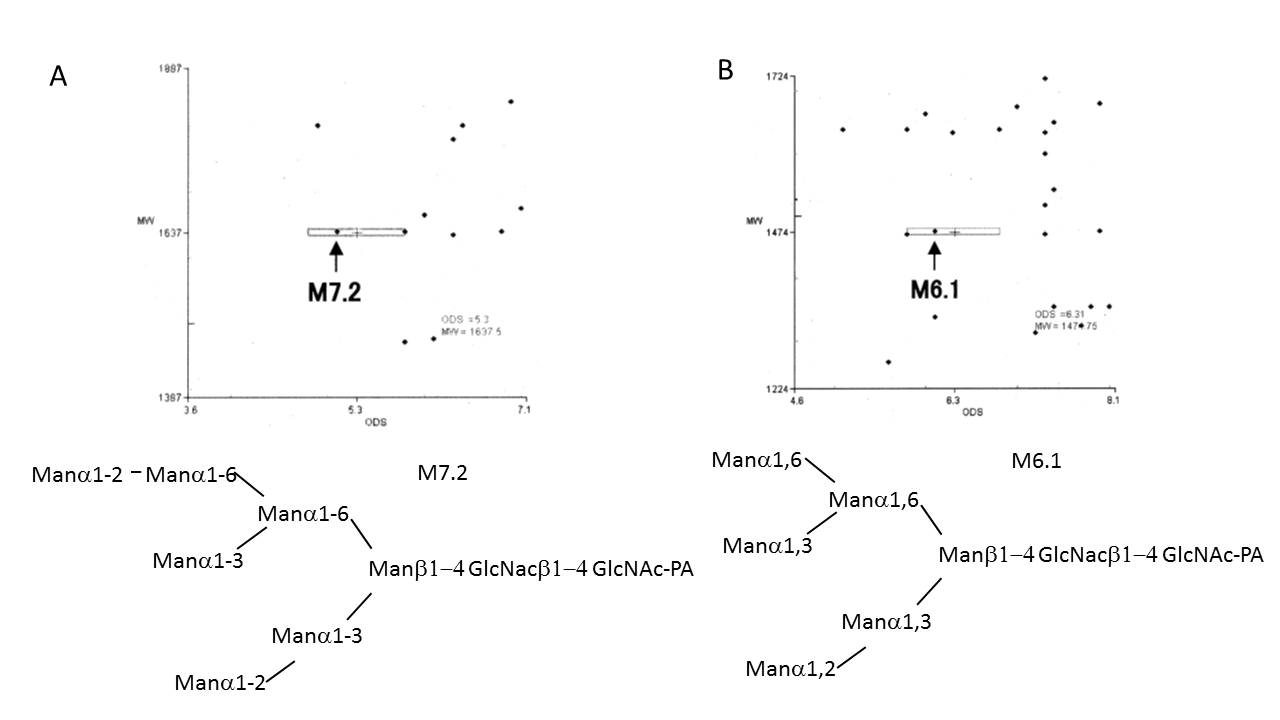


**Fig. S7** The structure of each *N*-glycan was estimated using GALAXY. (A) Estimated structure of N1. Upper panel: sample (GU from the ODS column: 5.3; GU from the Amide column: 7.8; and MW: 1683 Da) and candidate *N*-glycan estimated using GALAXY (GU from the ODS column: 5.1; GU from the Amide column: 8.1; MW: 1638 Da). (B) Estimated structure of N2. Upper panel: sample (GU from the ODS column: 6.3; GU from the Amide column: 6.9; and MW: 1475 Da) and candidate *N*-glycan estimated from GALAXY (GU from the ODS column: 6.1; GU from the Amide column: 7.1; MW: 1475 Da).

(C) Estimated structure of N3. Upper panel: sample (GU from the ODS column: 7.2; GU from the Amide column: 6.0; and MW: 1313 Da) and candidate N-glycan estimated from GALAXY (GU from the ODS column: 7.2; GU from the Amide column: 6.2; MW: 1313 Da). (D) Estimated structure of N4-1. Upper panel: sample (GU from the ODS column: 7.6; GU from the Amide column: 4.2, and MW: 989 Da) and candidate *N*
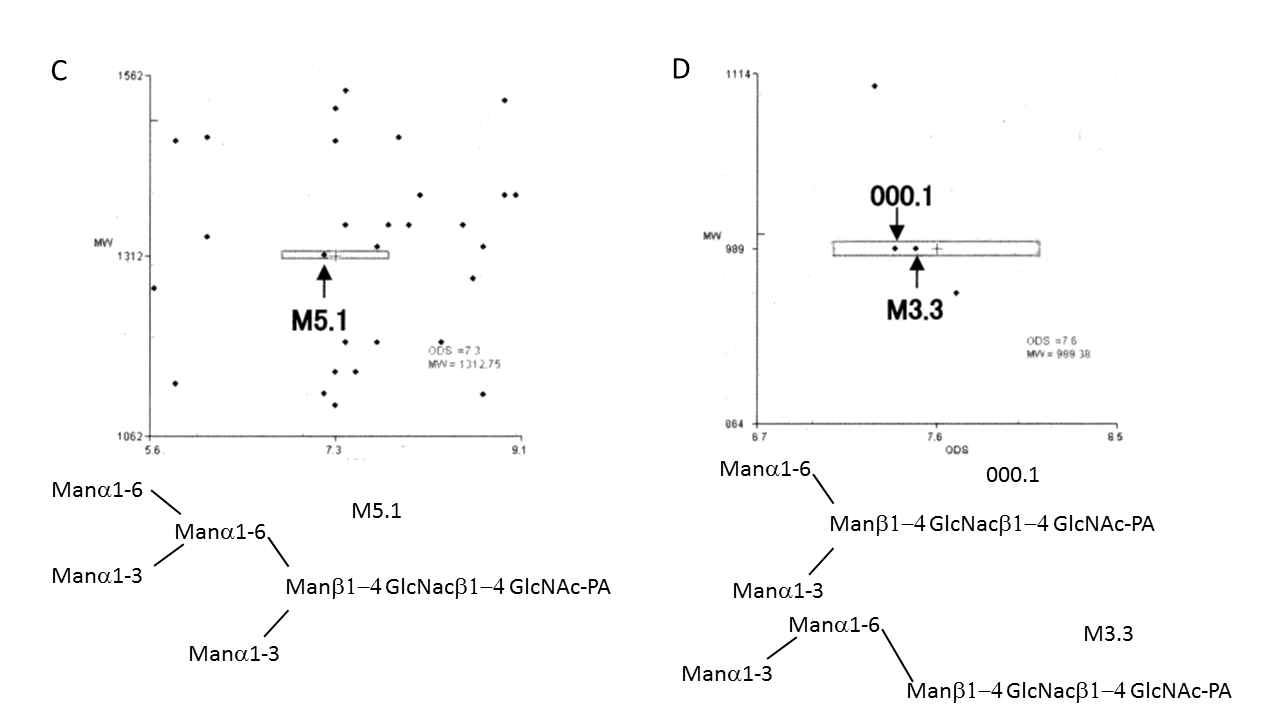
-glycan estimated from GALAXY (GU from the ODS column: 7.5; GU from the Amide column: 4.1, MW: 989 Da).


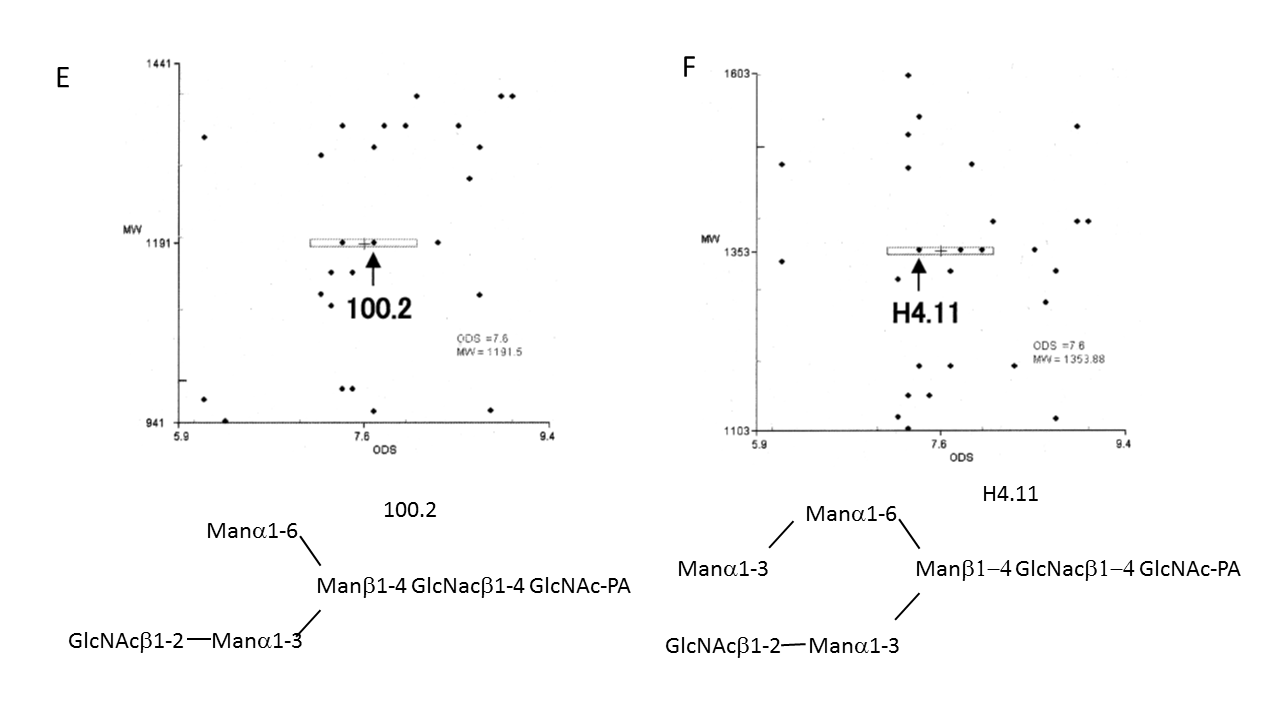


(E) Estimated structure of N4-2. Upper panel: sample (GU from the ODS column: 7.6; GU from the Amide column: 4.5, and MW: 1192 Da) and candidate *N*-glycan estimated from GALAXY (GU from the ODS column: 7.7; GU from the Amide column: 4.7; MW: 1192 Da). (F) Estimated structure of N4-3. Upper panel: sample (GU from the ODS column: 7.6; GU from the Amide column: 5.3, and MW: 1354 Da) and candidate *N*-glycan estimated from GALAXY (GU from the ODS column: 7.4; GU from the Amide column: 5.3, MW: 1354 Da).


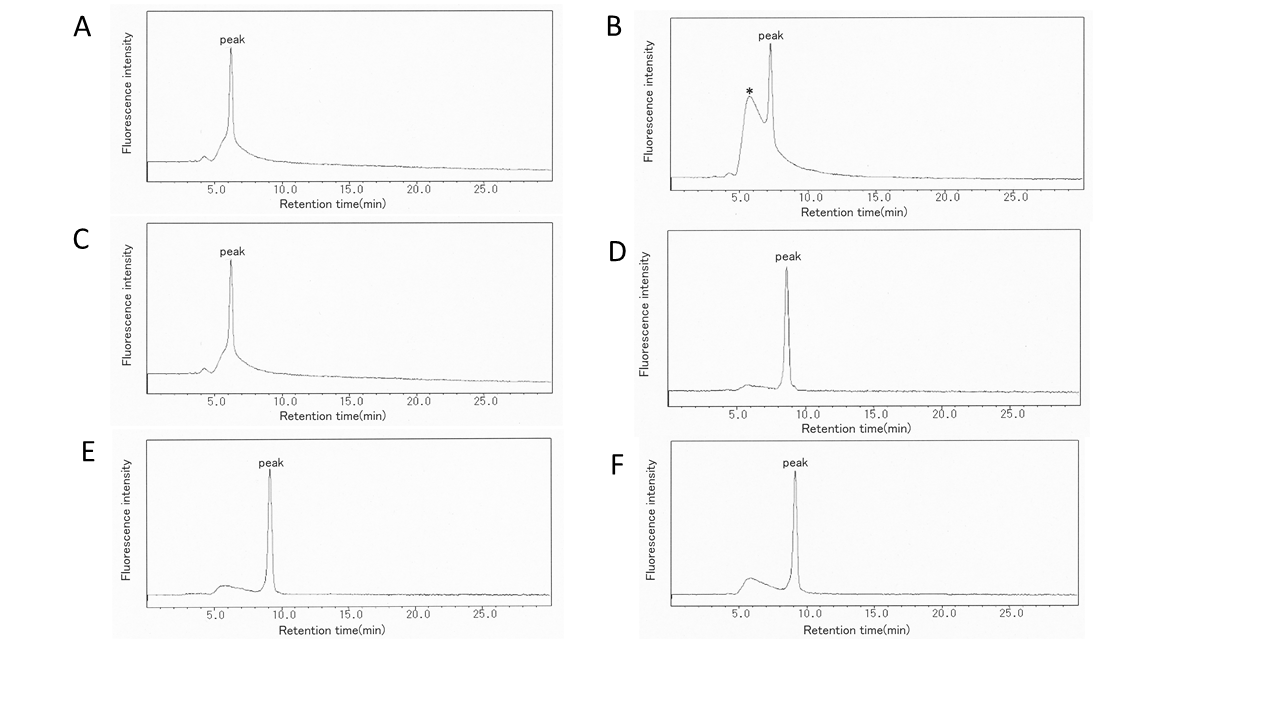


**Fig. S8** Co-injection of the sample and candidate reference PA-glycan. A, Peak N1; B, Peak N2; C, Peak N3; D, Peak N4-1; E, Peak N4-2; and F, Peak N4-3. The identity of each of the six peaks was confirmed.


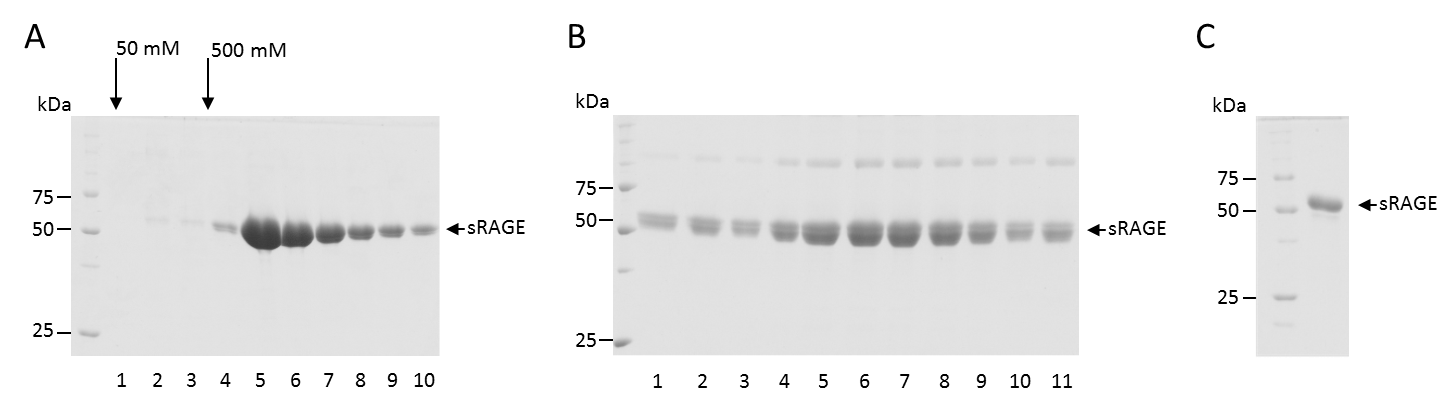


**Fig. S9** Purification of biotinylated sRAGE from MSGs extract. A, SDS-PAGE of TALON resin eluted fractions. The crude extract was cleared by centrifugation, and cleared lysates were gently mixed with TALON resin slurry for 15 min at 4°C. The resin was washed with 10 mM imidazole and packed into a column, and eluted with step wise manner (PBS containing50 mM or 500mM of imidazole). Lane 5-10 were collected, dialyzed against PBS, and the solution was used as TALON-purified fraction. B, SDS-PAGE of Mutein eluted fraction. 50% Mutein slurry (equilibrated PBS) was added to TALON-purified fraction (1ml Mutein slurry / 2 mg of TALON-purified protein) and gently mixed for 15 min at 4°C. The slurry was then packed onto the column, and the column was washed with PBS. RAGE fragments were eluted with a linear gradient of d-biotin (0–1.5 mM) by FPLC. Fractions containing protein at the expected molecular masses (lanes 3-10) were collected and dialyzed against PBS. C, SDS-PAGE of purified biotinylated sRAGE. Mutein purified fraction was analyzed by SDS-PAGE.


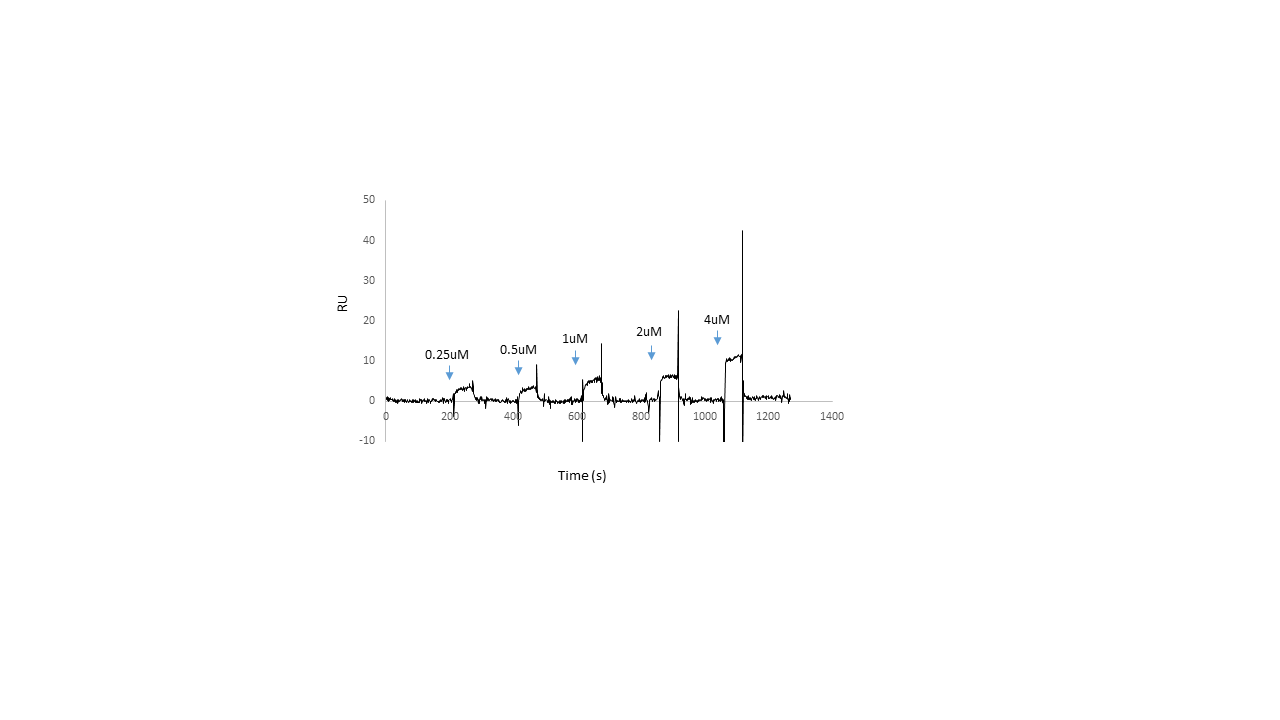


**Fig. S10** Surface plasmon resonance assay using the CM5 sensor chip. sRAGE without biotinylation was immobilized on a CM5 sensor chip, and surface plasmon resonance was performed.
